# Hemophagocytic Lymphohistiocytosis Associated with Scrub Typhus: Systematic Review and Comparison between Pediatric and Adult Cases

**DOI:** 10.3390/tropicalmed3010019

**Published:** 2018-02-14

**Authors:** Tameto Naoi, Mitsuya Morita, Tadataka Kawakami, Shigeru Fujimoto

**Affiliations:** 1 Stroke Center, Jichi Medical University 3311-1, Yakushiji, Shimotsuke-city, Tochigi 329-0498, Japan; shigeruf830@jichi.ac.jp; 2 Rehabilitation Center, Jichi Medical University 3311-1, Yakushiji, Shimotsuke-city, Tochigi 329-0498, Japan; morita@jichi.ac.jp; 3 Division of Neurology, Department of Internal Medicine, Shin-Oyama City Hospital 2251-1, Hitotonoya, Oyama-city, Tochigi 323-0827, Japan; jms79023@jichi.ac.jp; 4 Division of Neurology, Department of Medicine, Jichi Medical University 3311-1, Yakushiji, Shimotsuke-city, Tochigi 329-0498, Japan

**Keywords:** scrub typhus, *Orientia tsutsugamushi*, tsutsugamushi disease, hemophagocytosis, hemophagocytic lymphohistiocytosis

## Abstract

Background: Scrub typhus is a mite-borne bacterial infection caused by *Orientia tsutsugamushi*. Hemophagocytic lymphohistiocytosis (HLH) is a potential severe complication. Most reported cases of HLH associated with scrub typhus were single cases or case series with a small sample sizes. Thus, no clear consensus exists on clinical manifestations and differences between pediatric and adult cases of this condition. Methods: a systematic search of English and Japanese articles from PubMed, PubMed Central, and Directory of Open Access Journals databases was performed from 3 December 2016 to 28 December 2017. The primary outcome was mortality in patients with HLH associated with scrub typhus; secondary outcomes were differences in clinical symptoms, laboratory findings, and treatment between pediatric and adult patients with HLH associated with scrub typhus. Results: thirty cases of HLH associated with scrub typhus were identified (age range: 2 months to 75 years; median age: 21.5 years, male:female ratio, 1:1). Eschar was frequently observed in the pediatric group (*p* = 0.017), whereas acute kidney injury was more prevalent in the adult group (*p* = 0.010). Two patients died of intracranial hemorrhage complicated with multiple organ failure; overall mortality rate was 6.7%. Conclusions: HLH associated with scrub typhus could be cured with remarkable improvement using single antibiotic therapy in approximately half the cases, with the mortality rate being relatively lower than that of HLH associated with other secondary causes.

## 1. Introduction

Scrub typhus is a mite-borne bacterial infection caused by *Orientia tsutsugamushi* [1]. After the incubation period of 6–21 days, the onset of this disease is characterized by the presence of fever, headache, skin rash, myalgia, cough, and gastrointestinal conditions [1,2]. Presence of an eschar is the most crucial and distinguishing clinical manifestation of scrub typhus. The clinical course of scrub typhus is usually mild and self-limiting, with the patient spontaneously recovering after a few days. However, scrub typhus, especially in misdiagnosed cases due to overlooked/absent eschars, may progress into focal or disseminated multiorgan vasculitis [3,4,5,6]. Symptoms worsen and patients deteriorate within several days or weeks. [7,8]. Diagnosis relies on clinical suspicion, which should lead to appropriate laboratory investigations being performed. Failure to correctly diagnose scrub typhus can lead to ineffective empirical antibiotic therapy with beta-lactam-based regimens [9].

Hemophagocytic lymphohistiocytosis (HLH) is a critical systemic inflammatory condition that develops as a result of cytokine overproduction [10,11]. HLH is classified according to the causative factor involved and is categorized into genetic HLH—which occurs in infants—and secondary HLH—which is encountered regardless of age. Secondary HLH is mainly triggered by malignant lymphoma, viral and bacterial infections, and collagen diseases. HLH is a potential severe complication of scrub typhus, with an increasing number of cases reported in the last 10 years. However, most reported cases have been either single cases or case series with a small sample size. Thus, clinical manifestations and outcomes of patients with HLH associated with scrub typhus have not been well described. The dynamics of cytokines differs between children and adults [12]. In addition, most clinical guidelines, prospective studies, and treatment trials to date have focused on pediatric patients [11]. Therefore, there might be differences in clinical characteristics and treatment between pediatric and adult patients with HLH associated with scrub typhus. Here we present a systematic review of HLH associated with scrub typhus and compare the condition between pediatric and adult cases.

## 2. Methods

### 2.1. Search Strategy and Study Selection

A systematic search of English and Japanese articles from PubMed, PubMed Central, and Directory of Open Access Journals databases without year limitation was performed from 3 December 2016 to 28 December 2017. The following terminologies were used for the data search: hemophagocytic lymphohistiocytosis or hemophagocytosis or erythrophagocytosis or macrophage activation syndrome AND scrub typhus or *Orientia tsutsugamushi* or tsutsugamushi disease. The references of all articles were crosschecked for relevant articles. The diagnosis of scrub typhus was defined based on individual studies, including those with no eschar but with at least one positive laboratory diagnostic test, namely IgM antibody against *O. tsutsugamushi*, Weil–Felix test, or polymerase chain reaction in the acute phase. The Weil–Felix test lacks specificity and sensitivity but is a rapid diagnostic tool. The diagnosis of HLH was based on the histopathology of hemophagocytosis found in the bone marrow or on the fulfillment of the HLH-2004 diagnostic criteria. Studies were included if they provided detailed clinical data for patients with HLH associated with scrub typhus. The following exclusion criteria were applied: (1) Autopsy cases without their clinical course. (2) Journals in languages other than English and Japanese. (3) Patients diagnosed with scrub typhus but suspected of other coinfections triggering HLH. The literature search was performed according to the Preferred Reporting Items for Systematic Reviews and Meta-Analyses (PRISMA) statement recommendations. 

### 2.2. Data Extraction

Extracted data were as follows: year of study, country, patient’s clinical symptoms and signs, laboratory findings, diagnostic test, treatment, and outcomes. Extracted data were divided into pediatric (age <19 years) and adult (age ≥19 years) groups. 

### 2.3. Outcome Measures

The primary outcome was mortality in patients with HLH associated with scrub typhus, and secondary outcomes were the differences in the clinical symptoms, laboratory findings, and treatment between pediatric and adult patients with HLH associated with scrub typhus. 

### 2.4. Statistical Analysis

The categorical variables were compared using Fisher’s exact and Chi-square tests, and *p* <0.05 was considered statistically significant. 

## 3. Results

**Figure 1:**
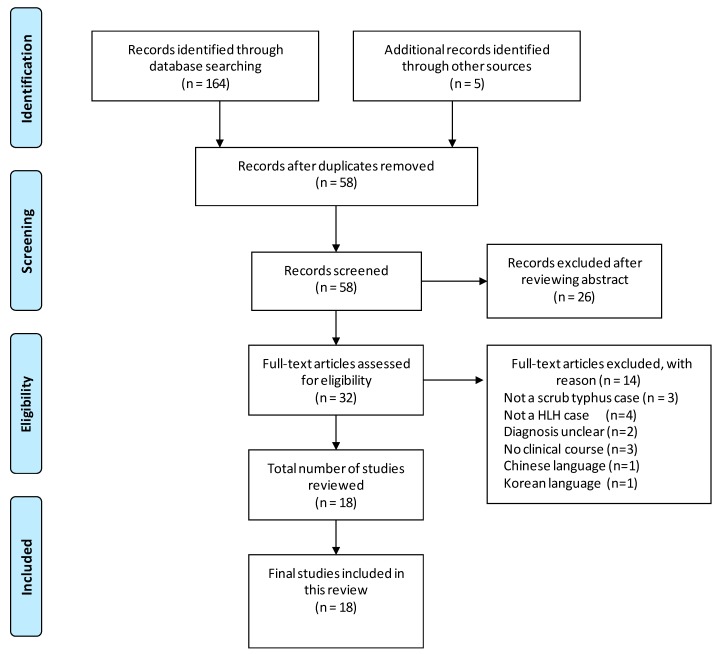
PRISMA flow chart showing the selection of studies for the review.

A total of eighteen articles on HLH associated with scrub typhus were identified (Figure 1). Twenty-six articles were excluded after reviewing the abstract as alternative diagnosis or basic research. Fourteen articles were excluded, and the complete details of the reason behind article exclusion are displayed in Figure 1. 

### 3.1. Diagnosis of Scrub Typhus and HLH

A total of 18 articles describing 30 cases of HLH associated with scrub typhus were identified (age range: 2 months to 75 years; median age: 21.5 years), belonging to diverse nationalities, including India (10 cases, 33.3%), China (9 cases, 30%), Japan (6 cases, 20%), South Korea (2 cases, 6.7%), Taiwan (2 cases, 6.7%), and Sri Lanka (1 case, 3.3%) [13,14,15,16,17,18,19,20,21,22,23,24,25,26,27,28,29,30]. Timely diagnosis includes the search for an eschar, which was detected in 20 of the 30 cases (66.7%). Table 1 summarises the clinical characteristics of all reported cases. Table 2 shows the clinical course of each patient. Mean days of illness prior to admission was 9.7 days in adult (n = 12) and 7.7 days in pediatric (n = 13) patients. HLH was diagnosed on the basis of the pathology of hemophagocytosis, including macrophage phagocytosis of the bone marrow or conformance with the HLH-2004 diagnostic criteria (Table 3, [10]). Bone marrow biopsy was performed in 29 cases and histological evidence for hemophagocytosis was observed in 28 cases. One patient was noted to be negative for hemophagocytosis on histological examination but nevertheless conformed with the HLH-2004 diagnostic criteria (case 21). One case lacked any record of bone marrow biopsy (case 15), although the HLH-2004 diagnostic criteria were met. Molecular analysis for primary HLH was performed in two cases (cases 14 and 15), but no genetic abnormality was detected in either case.

### 3.2. Clinical Findings of HLH Associated with Scrub Typhus

The subjects were divided into two groups: pediatric (n = 13, age <19 years, median age 5.0 years) and adult (n = 17, age ≥19 years, median age 47.0 years). The clinical manifestations of this disease are summarized in Table 4. The cardinal symptoms in both the groups included high fever and conditions involving enlarged lymphohematopoietic organs, such as lymphadenopathy and hepatosplenomegaly. Other organ involvements were primarily observed in the pulmonary and central nervous systems. The eschar was significantly more frequently observed in the pediatric group (*p* = 0.017), whereas acute kidney injury (AKI) was significantly more prevalent in the adult group (*p* = 0.010). Occurrence of acute respiratory distress syndrome (ARDS) was relatively frequent in the pediatric group, and more than half of the pediatric patients required invasive artificial ventilation. Among all 30 patients, two (cases 16 and 25) died of intracranial hemorrhage complicated with multiple organ failure, with an overall mortality rate of 6.7%. One patient survived with permanent neurological sequela (case 14). The remaining 27 patients survived or were cured. Neurological presentations were heterogeneous and included seizure, consciousness disturbance, intracranial hemorrhage, altered sensorium, meningitis, and encephalomyelitis.

### 3.3. Laboratory Findings of HLH Associated with Scrub Typhus

The laboratory findings are summarized in Table 5. More than half of the cases from each of the groups demonstrated anemia, thrombocytopenia, and liver dysfunction. Soluble interleukin 2 receptor (sIL2R) was examined in only two patients (cases 4, 16) despite the fact that HLH-2004 diagnostic criteria include the sIL2R level. Natural killer cell activity was evaluated in four patients (cases 14, 22–24). Not all laboratory tests were conducted and reported in the cases reviewed. Thus, sample size was small and no significant difference among their laboratory findings was noted.

### 3.4. Treatment of HLH Associated with Scrub Typhus

The treatment regimen for HLH associated with scrub typhus can be broadly classified into two categories: antibiotics used to treat scrub typhus and additional therapy (Table 1). Antibiotics such as doxycycline (18/30, 60%), minocycline (6/30, 20%), chloramphenicol (3/30, 10%), azithromycin (2/30, 6.7%), and clarithromycin (1/30, 3.3%) were applied as therapeutics. Additional therapy was significantly more frequently provided in the pediatric group (9/13, 69.2%) than in the adult group (4/17, 23.5%), (*p* = 0.012). The additional therapies for HLH included dexamethasone therapy (2/30, 6.7%), etoposide chemotherapy (3/30, 10%), cyclosporine (1/30, 3.3%), and intrathecal methotrexate (1/30, 3.3%). Intravenous immunoglobulin (IVIG) was used in 7/30 (23.3%) patients. 

## 4. Discussion

We performed a systematic review of reported cases of patients with HLH associated with scrub typhus and compared the differences in their clinical manifestation between the pediatric and adult groups. HLH associated with scrub typhus could be cured with remarkable improvement using single antibiotic therapy in approximately half the cases. The overall mortality rate was 6.7%, and the clinical outcome in HLH associated with scrub typhus was found to compare relatively well with that in HLH associated with other secondary causes. In comparison, HLH cases associated with Epstein–Barr virus (EBV) have been reported to have an early death rate of 14.1%, mostly owing to hemorrhage and infection [31]. Other studies reported that 24.2% patients with HLH associated with EBV died within 2 months of hospitalization [32]. Mortality rates were approximately 50% in patients with HLH associated with tuberculosis [33] and 52.1% in adult patients with HLH secondary to miscellaneous diseases [34]. 

In this review, the eschar was less likely to be found in adult patients. As per a previous study, the absence of eschar is an independent predictive risk factor for the fatal outcome in patients with scrub typhus [35]. In this review, however, the correlation between overlooked/absent eschar and HLH condition was not clarified. The rate of existence of eschar differs across the endemic region, with previous reports of 87% in Japan [36], 78.9% in Korea [37], and <10% in Thailand [38] and India [39]. Although there is no clear explanation for the higher frequency of eschar in the pediatric cases, variation in eschar incidence could be related to the *O. tsutsugamushi* strain, bacterial load in the blood, and host immunity [38]. 

The clinical manifestations of HLH associated with scrub typhus were diverse and non-specific, but the involvement of pulmonary and neurological aspects as well as that of the hematological disorder and coagulopathy was evident. Typical ARDS is a relatively lethal and disabling syndrome [40]. In contrast, in this review, ARDS was frequently observed in pediatric cases, with a positive response to treatment. The hallmark of pathophysiology in ARDS is the loss of the alveolar epithelium—endothelial barrier function under the condition of dysregulated coagulation and the overproduction of inflammatory factors such as IL-6 and IL-8 [40]. There is no clear explanation for the higher frequency of ARDS in the pediatric group; however, clinical and animal studies suggest age-dependent differences in the mechanism of ARDS [41]. Involvement of CNS resulted in severe outcomes, leading to the death of two patients (cases 16 and 25), and one patient experienced permanent neurological sequela (case 14). Neurological involvements were frequently observed, although the magnetic resonance imaging (MRI) finding was evident in only one patient (case 14). As reported in a previous case study, the CNS involvement of scrub typhus was associated with essentially normal MRI [42]. Autopsy studies revealed that leptomeningitis and vasculitis of the capillaries, arterioles, and small arteries within CNS are the main pathological processes reported [3,43]. Vasculitis, thrombocytopenia, and coagulopathy may result in fatal intracranial hemorrhage. Older age predicts AKI among patients with scrub typhus [44]. In patients with scrub typhus, the mechanism of AKI is considered to be impaired renal perfusion due to volume loss or increased vascular permeability [45]. Other potential mechanisms include direct tubular toxicity causing acute tubular necrosis, interstitial nephritis, and thrombotic microangiopathy secondary to disseminated intravascular coagulopathy (DIC). 

Anemia, thrombocytopenia, and other laboratory findings in patients with scrub typhus lead to occasional deterioration within a few weeks [8,13,18,26], which suggests that delayed treatment meets the HLH-2004 diagnostic criteria. However, there is no evidence so far to show that prompt treatment averts HLH. In fact, Takami et al. [17] reported two patients (cases 5 and 6) who showed hemophagocytosis on bone marrow aspiration without anemia, thrombocytopenia, or elevated liver dysfunction. Their studies revealed that scrub typhus triggers hemophagocytosis in the early stage of the disease. 

A total of 16 of the 30 cases (53.3%) were successfully treated with a single antibiotic therapy. Single antibiotic therapy resulted in rapid clinical improvement. However, it is challenging to explain this rapid improvement based on the bacteriostatic actions of these antibiotics. In fact, *O. tsutsugamushi* DNA is generally detected in the peripheral blood during the recovery phase of patients with scrub typhus [46]. Minocycline and doxycycline modulate cytokine levels and possess anti-inflammatory effects [47,48]. The anti-inflammatory functions of these antibiotics may be somewhat responsible for the rapid defervescence. However, treatment with chloramphenicol, which has no evidence supporting the anti-inflammatory properties, also exhibited the same rapid response. Therefore, the mechanism of rapid defervescence in patients with scrub typhus remains to be elucidated. 

In tandem with the diagnostic process, the treatment protocols for HLH associated with scrub typhus were extrapolated from the HLH-2004 treatment protocols [10]. However, the application of HLH-2004 treatment protocols to HLH associated with scrub typhus is poorly understood. A couple of patients were initially suspected to have a blood disorder because of pancytopenia and DIC. In these patients (cases 1 and 14), bone marrow aspirations were performed to confirm the HLH condition prior to diagnosing scrub typhus, and additional therapies were initiated antecedent to the antibiotics. The clinical course deteriorated in patient number 14. The other patient (case 1) did not show defervescence with oral prednisolone; instead, after starting minocycline the next day, this patient’s fever subsided. Some patients were treated with IVIG as immunomodulatory therapy. IVIG therapy has been successfully used in adults with HLH associated with different causes, although no randomized, controlled clinical trial has investigated IVIG therapy for HLH [11]. 

The present review has certain limitations. There was heterogeneity of diagnostic methods and data collected among the studies included in this review, indicating that the meaningful analysis of the data was limited. Only those cases that were reported in English and Japanese languages were selected; thus, the number of cases became fewer, and the significant differences in some clinical signs and laboratory findings remained unclear. Second, this review represents an incomplete picture of HLH associated with scrub typhus. The studies included those conducted in 6 countries, and reports from other countries in Southeast, South, and Central Asia as well as Pacific islands were lacking. This may be due to the lack of awareness or diagnostic limitations regarding HLH in other settings. Third, laboratory findings in some reported cases, for example, the sIL2R and ferritin levels, showed the measurement upper limit because of the limit of each facility’s ability. Therefore, the correlation between the clinical manifestations and laboratory findings was not always precise. 

## 5. Conclusions

Rapid defervescence was noted in patients with HLH associated with scrub typhus, although much remains unknown. The mortality rate of this condition is relatively lower than that of HLH associated with other pathological conditions. Thus, we recommend that clinicians should be aware of the local epidemiology of scrub typhus and the potential for patients to develop HLH as a complication. They should seek to diagnose and treat patients promptly. 

## Figures and Tables

**Table 1:** Cases of HLH associated with scrub typhus

Year (Reference)	Case No.	Age/Sex	Country	Eschar Location	Diagnostic Test for Scrub Typhus	HPS Findings in BM	HLH Criteria 2004	Outcome
1992 [13]	1	47/male	Japan	ND	IFA; Gilliam	+	ND	survived/cured
1994 [14]	2	53/female	Japan	back	IFA; Gilliam	+	ND	survived/cured
2000 [15]	3	21/male	Taiwan	ND	Weil–Felix; OX-K	+	ND	survived/cured
2001 [16]	4	53/female	Japan	left thigh	IFA; Karp	+	ND	survived/cured
2002 [17]	5	75/female	Japan	left buttock	IFA; Gilliam	+	ND	survived/cured
	6	69/female	Japan	left leg	IFA; Gilliam	+	ND	survived/cured
2006 [18]	7	74/female	Japan	external genitalia	IFA; Karp, Kato, Gilliam	+	ND	survived/cured
2009 [19]	8	58/female	Sri Lanka	perineum	IgG IFA	+	ND	survived/cured
2010 [20]	9	22/male	India	not detected	Weil–Felix; OX-K, IgM ELISA	+	ND	survived/cured
2010 [21]	10	35/male	India	posterior chest wall	IgM ELISA	+	ND	survived/cured
	11	61/male	India	ND	IgM ELISA	+	ND	survived/cured
	12	23/male	India	ND	IgM ELISA	+	ND	survived/cured
2011 [22]	13	5/female	India	hypogastrium	Weil–Felix; OX-K, IgM	+	criteria not met	survived/cured
2012 [23]	14	9/female	South Korea	scalp	IgM IFA	+	criteria is met	survived with sequela
2013 [24]	15	8 months/male	South Korea	right inguinal area	IgM IHA	ND	criteria is met	survived/cured
2014 [25]	16	34/female	Taiwan	not detected	Weil–Felix; OX-K, IgM, PCR	+	criteria is met	died
2014 [26]	17	40/female	India	ND	Weil–Felix; OX-K	+	criteria is met	survived/cured
2015 [27]	18	19/female	India	not detected	IgM ELISA	+	criteria is met	survived/cured
	19	64/male	India	not detected	IgM ELISA	+	criteria is met	survived/cured
	20	45/male	India	left groin	IgM ELISA	+	criteria is met	survived/cured
2015 [28]	21	2 months/male	India	not detected	IgM ELISA	not observed	criteria is met	survived/cured
2016 [29]	22	6/male	China	left shoulder	Weil–Felix; OX-K	+	criteria is met	survived/cured
	23	4/female	China	right opisthotic area	Weil–Felix; OX-K	+	criteria is met	survived/cured
	24	3/female	China	left forearm	Weil–Felix; OX-K	+	criteria is met	survived/cured
2016 [30]	25	8 months/male	China	+ (location not described)	Weil–Felix and/or IgM IFA	+	criteria is met	died
	26	1/female	China	+ (location not described)	Weil–Felix and/or IgM IFA	+	criteria is met	survived/cured
	27	7/male	China	+ (location not described)	Weil–Felix and/or IgM IFA	+	criteria is met	survived/cured
	28	7/female	China	+ (location not described)	Weil–Felix and/or IgM IFA	+	criteria is met	survived/cured
	29	11/male	China	+ (location not described)	Weil–Felix and/or IgM IFA	+	criteria is met	survived/cured
	30	7/male	China	+ (location not described)	Weil–Felix and/or IgM IFA	+	criteria is met	survived/cured

BM: bone marrow, IFA: immunofluorescence assay, IgM: immunoglobulin M, IHA: indirect hemagglutination HPS: hemophagocytosis, ND: not described.

**Table 2:** Clinical course of patients with scrub typhus-associated HLH

Case No.	Age/Sex	Illness Days Prior to Admission	Illness Days Prior to Antibiotics	Illness Days Prior to Additional Treatment	Days to Confirm HLH	Treatment		Fever Subsided after Initiation Antibiotics
						Antibiotics	Additional treatment	
1	47/male	11 days	13 days	12 days	11 days	minocycline	PSL	within 24 h
2	53/female	ND	ND	not treated	ND	minocycline		within 72 h
3	21/male	14 days	ND	not treated	ND	doxycycline		within 24 h
4	53/female	8 days	8 days	not treated	8 days	minocycline		within 72 h
5	75/female	ND	ND	ND	ND	doxycycline	PSL	within 72 h
6	69/female	7 days	ND	not treated	ND	minocycline		ND
7	74/female	4 days	7 day	not treated	ND	minocycline		within 24 h
8	58/female	10 days	24 days	not treated	ND	doxycycline		within 72 h
9	22/male	10 days	12 days	not treated	ND	doxycycline		within 72 h
10	35/male	10 days	ND	not treated	ND	doxycycline		within 96 h
11	61/male	20 days	ND	not treated	ND	doxycycline		within 72 h
12	23/male	5 days	ND	not treated	ND	doxycycline		within 48 h
13	5/female	6 days	7 days	not treated	ND	doxycycline		within 48 h
14	9/female	7 days	18 days	9 days	9 days	(roxithromycin)	DEX, cyclosporine, etoposide	after 96 h
						doxycycline	intrathecal methotrexate	
15	8 months/male	10 days	10 days	ND	ND	clarithromycin	DEX, etoposide	within 96 h
16	34/female	7 days	7 days	not treated	ND	minocycline		(died)
17	40/female	10 days	ND	ND	ND	doxycycline	mPSL	ND
18	19/female	ND	ND	ND	ND	doxycycline	corticosteroid, etoposide	ND
19	64/male	ND	ND	ND	ND	doxycycline		ND
20	45/male	ND	ND	ND	ND	doxycycline		ND
21	2 months/male	5 days	9 days	9 days	9 days	doxycycline	IVIG	within 24 h
22	6/male	7 days	7 days	not treated	ND	chloramphenicol		within 24 h
23	4/female	9 days	9 days	not treated	ND	chloramphenicol		within 48 h
24	3/female	8 days	8 days	not treated	ND	chloramphenicol		within 24 h
25	8 months/male	9 days	ND	ND	ND	azithromycin	IVIG/mPSL	(died)
26	1/female	4 days	ND	ND	ND	azithromycin	IVIG/mPSL	ND
27	7/male	12 days	ND	ND	ND	doxycycline	IVIG/mPSL	ND
28	7/female	9 days	ND	ND	ND	doxycycline	IVIG/mPSL	ND
29	11/male	7 days	ND	ND	ND	doxycycline	IVIG	ND
30	7/male	7 days	ND	ND	ND	doxycycline	IVIG/mPSL	ND

DEX: dexamethasone, IVIG: intravenous immunoglobulin, mPSL: methylprednisolone, PSL: prednisolone, ND: not described.

**Table 3:** HLH-2004 diagnostic criteria

The diagnosis of HLH can be established if any one of two given factors is fulfilled:
1. A molecular diagnosis consistent with HLH
2. Diagnostic criteria for HLH are fulfilled (5 or more of 8 criteria below)*
Fever
Splenomegaly
Cytopenias (affecting ≥2 of 3 lineages in the peripheral blood)
Hemoglobin <90 g/L (in infants <4 weeks old; hemoglobin <100 g/L)
Platelets <100 × 10^9^/L
Neutrophils <1.0 × 10^9^/L
Hypertriglyceridemia and/or hypofibrinogenemia: fasting
Hypertriglyceridemia ≥3.0 mmol/L (i.e., ≥265 mg/dl), fibrinogen ≤1.5 g/L
Hemophagocytosis in the bone marrow, spleen, or lymph nodes
Low or absent natural killer cell activity (according to the local laboratory reference)
Ferritin ≥500 µg/L
Soluble CD25 (i.e., sIL2r) ≥2400 U/mL

* Supportive criteria include neurological symptoms, cerebrospinal fluid pleocytosis, conjugated hyperbilirubinemia and transaminitis, hypoalbuminemia, hyponatremia, elevated D-dimers, and lactate dehydrogenase. The absence of hemophagocytosis in the bone marrow does not exclude the diagnosis of HLH.

**Table 4:** Clinical manifestations

	Pediatric Group (n = 13)	Adult Group (n = 17)	*p*-value
Age range/median age	2 months–11 years/5.0 years	19–74 years/47.0 years	
Female	6/13 (46.2%)	9/17 (52.9%)	0.712
Mortality	1/13 (7.7%)	1/17 (5.9%)	0.844
Respiratory system			
ARDS	7/13 (53.8%)	4/17 (23.5%)	0.132
Pleural effusion	0/13 (0%)	1/17 (5.9%)	0.567
Pulmonary hemorrhage	1/13 (7.7%)	0/17 (0%)	0.433
Bronchitis	1/13 (7.7%)	0/17 (0%)	0.433
Invasive ventilator use	7/13 (53.8%)	5/17 (29.4%)	0.175
Central nervous system			
Seizure	3/13 (23.1%)	1/17 (5.9%)	0.290
Consciousness disturbance	0/13 (0%)	1/17 (5.9%)	0.567
Intracranial hemorrhage	1/13 (7.7%)	1/17 (5.9%)	1.000
Altered sensorium	1/13 (7.7%)	0/17 (0%)	0.433
Altered mental status	1/13 (7.7%)	0/17 (0%)	0.433
Meningitis	1/13 (7.7%)	0/17 (0%)	0.433
Encephalomyelitis	1/13 (7.7%)	0/17 (0%)	0.433
Gastrointestinal system			
Gall bladder distension	0/13 (0%)	1/17 (5.9%)	0.567
Abdominal pain	0/13 (0%)	2/17 (11.8%)	0.492
Other complication			
Fever	13/13 (100%)	17/17 (100%)	
Skin rash	9/13 (69.2%)	6/17 (35.3%)	0.065
Eschar	12/13 (92.3%)	8/17 (47.1%)	0.017
Splenomegaly ± hepatomegaly	11/13 (84.6%)	14/17 (82.4%)	1.000
Liver dysfunction	13/13 (100%)	15/17 (88.2%)	0.492
Lymphadenopathy	5/13 (38.5%)	8/17 (47.1%)	0.638
Acute kidney injury	0/13 (0%)	7/17 (41.2%)	0.010
Pedal edema	0/13 (0%)	1/17 (5.9%)	0.567
Tonsillar swelling	0/13 (0%)	2/17 (11.8%)	0.492
Myalgia	0/13 (0%)	2/17 (11.8%)	0.492
Leukemoid reaction	1/13 (7.7%)	0/17 (0%)	0.433
Hydrocele	1/13 (7.7%)	0/17 (0%)	0.433
Arthralgia	0/13 (0%)	1/17 (5.9%)	0.567

**Table 5:** Laboratory findings

	Pediatric Group (0–11 years)	Adult Group (≥19 years)	*p*-value
Hematological			
Hemoglobin <90 g/L	10/13 (76.9%)	10/15 (66.7%)	0.686
Platelets <100 × 10^9^/L	12/13 (92.3%)	14/17 (82.4%)	0.613
Neutrophils <1.0 × 10^9^/L	0/4 (0%)	6/12 (50%)	0.234
Coagulation			
Fibrinogen ≤1.5 g/L	9/12 (75%)	1/5 (20%)	0.101
Biochemical features			
Ferritin ≥500 µg/L	13/13 (100%)	11/13 (84.6%)	0.480
Triglycerides ≥265 mg/dl	9/11 (81.8%)	4/9 (44.4%)	0.160
Soluble CD25 (i.e., sil2r) ≥2400 U/ml	not examined	2/2 (100%)	
Low or absent natural killer cell activity	3/4 (75%)	not examined	
AST or ALT ≥50 IU/L	12/12 (100%)	13/17 (76.5%)	0.121
Creatinine ≥1.0 mg/dl	0/3 (0%)	5/10 (50%)	0.231
